# Exploring Fear of Cancer Recurrence (FCR) in cancer survivors from a medical social work perspective: A qualitative study of medical social workers in South Korea

**DOI:** 10.1371/journal.pone.0288059

**Published:** 2023-07-06

**Authors:** Ka Ryeong Bae, Yeojin Ahn, Joung Won Park, Seok-Joo Kim

**Affiliations:** 1 Department of Clinical Research Design & Evaluation, Samsung Advanced Institute for Health Sciences & Technology (SAIHST), Sungkyunkwan University, Seoul, South Korea; 2 Patient-Centered Outcomes Research Institute, Samsung Medical Center, Seoul, South Korea; 3 Division of Social Work, Samsung Medical Center, Seoul, South Korea; 4 Department of Social Welfare, College of Social Sciences, Daegu University, Gyeongbuk, South Korea; University of Sharjah College of Health Sciences, UNITED ARAB EMIRATES

## Abstract

**Background:**

Fear of Cancer Recurrence (FCR) in cancer survivors has been insufficiently addressed despite its imperativeness in cancer journey. Although several studies have investigated healthcare professionals’ experience with FCR in cancer survivors, a medical social work perspective has rarely been reflected. This study aimed to explore Korean medical social workers’ experience with intervening FCR in cancer survivors.

**Methods:**

Snowball sampling recruited 12 experienced medical social workers intervening with cancer survivors at tertiary or university cancer hospitals in South Korea. Individual and focus-group interviews (FGI) were conducted with the medical social workers. The interviews were recorded, transcribed, and analyzed by using an inductive qualitative content analysis.

**Results:**

Content analysis of the interviews extracted the following major themes regarding FCR in cancer survivors. First, when and how FCR among cancer survivors emerged at the early stage of medical social work interventions was identified. Second, how medical social workers dealt with FCR in cancer survivors was illustrated. Third, the responses of cancer survivors to medical social work interventions for FCR were assessed. Finally, the internal and external issues underlying the medical social work interventions for FCR among cancer survivors were revealed and discussed.

**Conclusion:**

Based on the results, this study suggested the implications on dealing with FCR in cancer survivors in the realm of medial social work profession. Furthermore, it expanded the discussion about FCR in cancer survivors from cancer hospitals to community.

## 1. Introduction

Fear of cancer recurrence (FCR) is a phenomenon defined as “fear, worry, or concern relating to the possibility that cancer will come back or progress” [1]. While its severity ranges, more than half of cancer survivors (58%) experience clinically severe FCR [2]. FCR is distinguished from hypochondriasis and other anxiety disorders because it is explicitly related to an actual diagnosis that could potentially lead to cancer recurrence [1, 3]. Severe FCR is associated with low quality of life [4] and can potentially trigger various symptoms that align with posttraumatic stress disorder [5]. Although FCR is one of the most critical issues among cancer survivors, it has been insufficiently addressed after cancer treatment [6].

Several qualitative studies exploring the phenomenon of FCR in cancer survivors have delivered practical knowledge of psycho-social interventions with cancer survivors [3, 7–10]. For example, one recent study found that cancer survivors seldom articulated the term “recurrence” or “fear of recurrence,” at regular outpatient visits with medical healthcare professionals such as oncologists and oncology nurses, which implies that they tend to share psychological stress or concerns with other non-medical healthcare professionals such as medical social workers or clinical psychologists [10]. Therefore, the knowledge and experience from non-medical healthcare professionals can contribute to enhancing the current comprehension of FCR and developing versatile strategies of addressing psycho-social issues among cancer survivors.

Among the various healthcare professionals, medical social workers in cancer hospitals are regarded as one of the key informants who can delineate the phenomenon of FCR among cancer survivors. The National Comprehensive Cancer Network (NCCN) distress guideline suggests that medical social workers are one of the primary providers of oncology psychosocial services [11, 12]. In their practice, medical social workers address a wide range of psycho-social issues and challenges that cancer survivors face during cancer journey [13]. Moreover, some cancer survivors feel comfortable sharing their concerns with medical social workers and are aware of the latter’s roles in addressing their psycho-social issues [14].

Despite its significance, a medical social work perspective has rarely been considered to address FCR in cancer survivors [4, 15]. Unfortunately, to date, few empirical studies have sought to clarify FCR in South Korea from this perspective. Therefore, this study aimed to illuminate the phenomenon of FCR in the context of medical social work and to discuss various issues and challenges of addressing FCR in cancer survivors.

## 2. Materials and methods

### 2.1. Study design

This study aimed to explore FCR in cancer survivors in terms of medical social work. Specifically, it focused on the professional experiences of medical social workers who worked with cancer survivors. Based on a qualitative research design, individual interviews and FGIs with medical social workers actively working with cancer survivors in tertiary or university cancer hospitals in South Korea.

This study was approved by the Institutional Review Board (IRB) of the Samsung Medical Center (IRB No. 2019-07-073). Prior to the interviews, all participants received detailed explanations of the interview procedures, confidentiality, data collection, and analysis. They were informed that they could voluntarily terminate the interview at any time. They also agreed that their interviews were recorded, transcribed, and stored encoded. All participants provided written informed consent for the study including audio recordings and received a small token of appreciation after the interview. For confidentiality reasons, the interviews were transcribed after assigning the participants a unique identification number.

### 2.2. Participants

This study established three eligibility criteria for the participants as follows: medical social workers who (1) were currently working at a tertiary or university cancer hospital in South Korea; (2) had at least 3 years of experience intervening (i.e., counseling, education, or research) with cancer survivors; and (3) agreed to participate into the study. Although the Ministry of Health and Welfare in South Korea estimated that, as of 2020, a total of 1,302 medical social workers worked at tertiary or general hospitals, the specific number serving cancer survivors was not identified [16]. Therefore, snowball sampling was used to recruit participants, who then recommended others who were eligible for the study. The interviews were conducted until data saturation was reached, and thus a total of 12 interviewees were recruited. Overall, the participants were homogeneous; most were women (N = 11), in their 30s (N = 6), and working in metropolitan areas. Nonetheless, the number of cancer survivors for whom the participants worked were heterogeneous (Table 1).

**Table 1:** Characteristics of the participants.

No	Gender	Age (year)	Length of career (year)	Type of interview	Region	Types of cancer survivors	Main job
01	Female	47	21	FGI (Group 1)	Metropolitan	Various cancer patients from low-income families	Counselling, Education
02	Female	32	8	FGI (Group 1)	Metropolitan	Lung, hematologic cancer patients	Counselling, Education, Research
03	Female	36	8	FGI (Group 1)	Metropolitan	Breast, gastric, colon cancer patients	Counselling, Education
04	Female	27	3	FGI (Group 1)	Metropolitan	Cancer patients before chemotherapy or surgery	Counselling, Education
05	Female	39	8	Individual interview	Province	Elderly patients, advanced cancer patients	Counselling
06	Female	28	5	FGI (Group 2)	Metropolitan	Various cancer patients	Counselling, Education
07	Female	45	15	FGI (Group 2)	Metropolitan	Gastrointestinal cancer patients	Counselling, Education, Research
08	Female	44	9	FGI (Group 2)	Metropolitan	Lung, gastrointestinal cancer patients	Counselling, Education
09	Female	41	16	FGI (Group 2)	Metropolitan	Lung, pancreatic cancer patients	Counselling
10	Female	36	6	Individual interview	Province	Breast cancer, hospice care patients	Counselling, Education, Research
11	Female	36	10	Individual interview	Province	Patients with newly diagnosed cancer	Counselling
12	Male	31	3	Individual interview	Province	Hospice & palliative cancer care	Counselling, Inform patients, Education

### 2.3. Data collection

Using a semi-structured questionnaire, authors KB, YA, and JP conducted individual interviews (4 participants) and FGIs (8 participants of 2 groups) from December 2020 to February 2021; all participants took part in a one-session interview (1 hour). Interviews were implemented sequentially. Specifically, the participants in the individual interviews recommended others for the next individual interviews and FGIs. As a result of the COVID-19 pandemic it was impossible to conduct face-to-face interviews, therefore, these were conducted via phone and online (i.e., Zoom) for individual interviews and FGIs, respectively. The interviews were scheduled at the participants’ convenience. Prior to the main interview, they completed an 11-item preliminary questionnaire including basic sociodemographic information (e.g., age, sex, career, job responsibilities, etc.). Upon completing the preliminary questionnaire, the interviewer received the participants’ permission to begin the main interview with a semi-structured questionnaire that was developed based on comprehensive literature reviews and feedbacks from colleagues. Specifically, the semi-structured questionnaire, which consisted of open-ended questions, covered a wide range of topic on FCR in cancer survivors: (1) professional experience with cancer survivors who expressed signs of FCR, (2) medical social work interventions for FCR in cancer survivors, and (3) issues or challenges in dealing with FCR in cancer survivors. In addition to the semi-structured questionnaire, the interviewer asked follow-up questions depending on the participants’ responses. Additional field notes were taken to observe the participants’ verbal and non-verbal expressions during the interviews.

### 2.4. Content analysis

The audio recordings of the interviews were transcribed and analyzed by using inductive qualitative content analysis, as proposed by Elo and Kyngäs [17]. First, KB and JP carefully noted the participants’ responses to the questions during the interviews, which enabled them to obtain insights into the overall interview themes. Second, KB and JP individually performed open (line-by-line) coding and conducted content analysis (i.e., categorization, tagging, and thematic analysis) as they meticulously and repeatedly read the transcripts. Third, KB and JP shared the tentative results of their analyses with each other and discussed any discrepancies and conflicts until a consensus was reached. Finally, all authors (i.e., KB, YA, JP, and SK) reviewed and finalized the results.

### 2.5. Trustworthiness of the information

Following the guidelines of Lincoln and Guba, this study established four criteria to validate the trustworthiness of the data collection and analysis: (1) credibility, (2) transferability, (3) dependability, and (4) confirmability [18].

First, the sampling method made it possible to ensure the credibility of the collected data. An important aspect of the study was to recruit the key informants who had extensively interacted with cancer survivors and could describe the psycho-social issues of cancer survivors. Accordingly, this study only recruited experienced medical social workers who actively worked with cancer survivors at least three years. Second, the transferability was established as the study data were collected and analyzed until no new information was discovered. The collected data covered an extensive range of information regarding medical social work for cancer survivors (i.e., the participants’ careers and workplaces, characteristics of cancer survivors with whom the participants had engaged, etc.). Third, the dependability of the study was verified through rigorous data collection and analysis. The interviews were recorded in detail, and all authors confirmed that the extracted results were consistent with the collected data. Finally, an audit trail analysis was performed to assure the confirmability of the results. In this study, the multidisciplinary professionals analyzed the data and audited the results. Specifically, two authors from oncological nursing and medical (cancer) social work analyzed the collected data and two authors from social work and psychology reviewed and verified the results.

### 2.6. Researcher training and preparation

This study was designed and conducted by the multidisciplinary professionals, including an oncology nurse, a medical (cancer) social worker, and a professor of social work. KB is an advanced oncology nurse and professor who teaches qualitative research methods at a graduate school, while JP is an experienced medical (cancer) social worker at a tertiary cancer hospital. SK is a professor of social work who lectures on research methodology and has conducted various community-based studies on physical and mental health. Prior to the study, all authors attended several workshops and seminars on qualitative research methods and studies on cancer survivors.

## 3. Results

In the in-depth interviews, the medical social workers discussed a variety of experiences regarding FCR in cancer survivors and interventions. Using content analysis, five major themes were extracted from the interviews and systematically organized according to the process of medical social work intervention as follows: (1) emergence of FCR in the preliminary stage, (2) medical social work interventions for FCR, (3) cancer survivors’ responses to medical social work interventions, (4) internal, and (5) external challenges in medical social work intervention for FCR.

### 3.1. Emergence of FCR in the preliminary stage

In the preliminary stage of the intervention, the medical social workers rarely captured explicit FCR among cancer survivors. However, they discovered manifested FCR in subsequent interactions with cancer survivors and identified a few characteristics of cancer survivors who were vulnerable in FCR.

#### 3.1.1. FCR coming up to the surface

FCR was an intangible topic in the early stages of medical social work interventions. Once cancer survivors established a rapport with a medical social worker, FCR became one of the dominant issues for which they often sought help in following sessions. As such, FCR was explicitly encountered by the medical social workers, particularly during in-depth counseling.

“Maybe it is because cancer survivors feel that they build a rapport with me after a few sessions… but they do not say anything about FCR in the beginning. It is in the later sessions that cancer survivors begin to open…” (Participant 06)

“There was one cancer survivor who was extremely worried about recurrence, always talking about fear of recurrence whenever visiting us or over the phone, and calling us three to four times a month…” (Participant 09)

“Because their psychological concerns emerged and developed during individual or group counseling, I feel like everyone that I met during counseling, like 10 out of 10, brought it up.” (Participant 01)

#### 3.1.2. Signals of FCR

Cancer survivors’ FCR was projected through various signals. Cancer survivors were less likely to articulate the terms, “recur” or “recurrence,” and instead verbalized implicit phrases that implied FCR such as “worried”, “ill again”, “go wrong”, etc.

“Few cancer survivors used the word ‘recur’ or ‘recurrence.’ They usually expressed their concerns through the phrases like ‘being ill again,’ ‘having another cancer,’ ‘cancer spreading,’ and so on " (Participant 11)

“Worried is the most commonly and frequently used word among cancer survivors when they show fear of reoccurrence.’” (Participant 2)

#### 3.1.3. Manifesting and elevated FCR

FCR among cancer survivors began to manifest at the end of active cancer treatment, especially when cancer survivors were uncertain about what to do afterwards. Moreover, cancer survivors expressed more explicit FCR when they noticed other people experiencing cancer recurrence or encountered family members’ concerns.

“We have several cancer survivors who were diagnosed with cancer 5–10 years ago. Even after this time, they still talk about their FCR. They seem to be more concerned or anxious when they see someone around them experiencing cancer recurrence…” (Participant 3)

Furthermore, the medical social workers also observed elevated FCR in various situations. For example, they often noticed elevated FCR when cancer survivors talked about physical discomfort (esp., unexpected pains at home) during phone calls. At cancer hospitals, the medical social workers usually recognized FCR when cancer survivors worried about their medical test results.

“Around the day of their medical test, (some cancer survivors say,) ‘I feel like my pain is getting worse. I am so scared of how the test will come out and it feels like my pain has gotten worse around the test day.’ I think that fears of the test results provoke them to talk more about FCR.” (Participant 07)

#### 3.1.4. Characteristics of cancer survivors expressing FCR

As the medical social workers interacted with many cancer survivors, they were able to identify the characteristics of those who actively expressed FCR at an early stage of intervention. These characteristics included age, sex, and caregiver responsibilities. For instance, FCR is more prominent among cancer survivors with Triple-Negative Breast Cancer (TNBC), a disease that is prevalent among young adults and has relatively high recurrence risk among various types of breast cancers [19–21]. Likewise, cancer survivors who had maintained a healthy lifestyle before diagnosis, such as non-smoking females with lung cancer or non-drinking people with liver cancer, were more likely to seek help with FCR.

“I found that cancer survivors who were younger, female, diagnosed with early-stage cancer, or diagnosed with poor prognosis cancer talked about FCR much more than others.” (Participant 05)

“I’ve seen many cases where younger female survivors, particularly those who had a family or a child to raise, expressed FCR more strongly than others… Especially, cancer survivors with TNBC showed the deepest fear of recurrence.” (Participant 01)

As the medical social workers engaged cancer survivors with financial hardship, they observed an ambivalent relationship between FCR and economic status. Interestingly, cancer survivors with both low and high economic status commonly showed FCR due to different reasons. On the one hand, financial issues were directly related to FCR among low-income cancer survivors, especially those with less familial or social support.

“Cancer survivors who were near or under the poverty line but were ineligible for social benefits showed more anxiety than others. They should take care of their families but at the same time they should take care of their livelihoods…” (Participant 01)

On the other hand, cancer survivors with a stable economic status also actively expressed FCR. Because they could afford to utilize various resources (e.g., self-help groups, educational programs, etc.), they were more exposed to cancer-related issues, including FCR.

“I met several cancer survivors who attended a self-help group on weekdays. This meant that they had affluent information and a lower financial burden. If cancer survivors have financial difficulties, they must work on weekdays. Because those without financial burdens could frequently come here (i.e., self-help groups and counseling), they could have more opportunities to talk about various issues including, financial burden and psychological stress.” (Participant 06)

### 3.2. Medical social work interventions for FCR

The medical social workers applied the professional values and skills to intervening in cancer survivors’ FCR. Considering the cancer journey, the medical social workers usually utilized basic counseling and coping skills training. Furthermore, they linked useful resources to cancer survivors with FCR.

#### 3.2.1. FCR matters to all cancer survivors

All of the medical social workers in this study agreed that FCR among cancer survivors should be dealt with, emphasizing that FCR should be assessed and addressed for all cancer survivors because it was intimately associated with all aspects of their lives.

“If I am asked, ‘Is it necessary to address FCR in cancer survivors?’ I can confidently reply, ‘Of course’ because FCR is an important part of their lives.” (Participant 03)

"It is important to deal with FCR. Feelings such as fear and trouble can affect all aspects of their lives, such as cancer treatment, daily routines, family relations, and other interpersonal relationships. So, FCR is something to be seriously considered.” (Participant 11)

#### 3.2.2. Basic counseling skills as a major tool

Because their major roles were to provide counseling to cancer survivors, the medical social workers actively utilized listening and sympathizing in their interventions. Furthermore, a few of them tried to assess the level of cancer survivors’ FCR using relevant measurements, which enabled them to progress to in-depth counseling.

“First, I carefully listened to the emotions and experiences that my clients (cancer survivors) told me. Then I put them at ease and helped them stabilize their emotions, saying, ‘It is natural to go through these emotions and react that way.’ I helped them clear the air, so they could feel at ease.” (Participant 02)

“It is important to share emotions with others when they are nervous. Hence, I often asked my clients (cancer survivors) whether they had anyone with whom to share their feelings. If they replied, ‘I have nobody,’ I told them, ‘I can be your person.’ I closely attended to their feelings and empathized with them so that they could fully vent their complicated feelings.” (Participant 10)

#### 3.2.3. Strength-based approach and coping skill training

In addition to the basic counseling mentioned above, the medical social workers provided further interventions to cancer survivors who experienced FCR. These interventions were grounded in cancer survivors’ strengths and behaviors (i.e., focusing on and summarizing problems, reinforcing their strengths, and guiding how to establish priorities and goals). Additionally, most of the medical social workers preferred to implement coping skills training rather than Cognitive Behavioral Therapy (CBT) because of their familiarity with the practice.

“I pay more attention to the positive sides of my clients (cancer survivors), rather than the negative ones. For example, I approached FCR as something to reinforce my clients’ strengths as I utilized solution-focused brief therapy and narrative therapy. Consequently, my interventions enabled cancer survivors to concentrate more on their issues and treatment.” (Participant 07)

“(Because a little bit of coping skills can significantly help to my clients), my clients (cancer survivors) and I tried to practice coping skills together. I encouraged them to practice their skills at home. When I was allowed to meet my clients several times, I monitored how well they followed and exercised the coping skills that I previously introduced to them.” (Participant 08)

#### 3.2.4. Informed itinerary on cancer treatment

The medical social workers suggested that mapping the cancer journey was effective in alleviating FCR when cancer survivors experienced obscurity or uncertainty after cancer treatment.

“I often met a few cancer survivors who showed FCR right after diagnosis. In this situation, it was very effective to explain the journey of cancer treatment. I explained the details of the cancer journey to them, saying, ‘You are going to receive these kinds of treatments. These challenges may be experienced during cancer treatment. If you face these challenges, it is more helpful for you to respond to them in these ways.” (Participant 04)

#### 3.2.5. Referrals or links to resources

When cancer survivors showed severe FCR or needed further attention, they were encouraged to contact their oncologists or were referred to a psychiatrist. If they needed to receive peer support or interact with other cancer survivors, the medical social workers found mentoring programs or self-help groups and linked cancer survivors to these resources.

“Although we could not exactly quantify my clients’ (cancer survivors’) severity of FCR in the counseling, we did refer them to other healthcare professionals when they persistently showed a certain level of fear that was assumed to be very serious.” (Participant 11)

“I saw that cancer survivors managed FCR as they met mentors (other cancer survivors).” (Participant 09)

### 3.3. Cancer survivor’s responses to medical social work interventions

The medical social workers reported that most cancer survivors with FCR had positive experiences with medical social work interventions. Throughout the medical social interventions, cancer survivors felt relieved, enhanced their understanding of FCR, and became more sensitive to it. However, their responses to the interventions varied according to their residence.

#### 3.3.1. Feeling relieved

The medical social workers found several cancer survivors whose psychological symptoms were relieved or resolved after counseling. In particular, cancer survivors who were nervous about unexpected symptoms or pain tended to feel more comfortable, both psychologically and physically, after counseling with the medical social workers.

“One client (cancer survivor) who had been very anxious in the counseling sessions called me the next day and said, ‘Oh, I do not need to meet you earlier (before the next counseling). The backache I had before is gone.’” (Participant 06)

#### 3.3.2. Realizing the nature of FCR

After counseling with medical social workers, many cancer survivors reflected that they did not try any methods to deal with their fears and worries. They also realized that their fears were not a big issue as they had originally pictured.

"As you know, there were a few cancer survivors who did nothing but worry all day. However, they admitted, ‘You asked me to try these skills, but I did none of them.’ Then they gradually became aware of something by themselves. They were able to understand or even regret that they had problems because they did nothing.” (Participant 02)

#### 3.3.3. Confidence in moving forward

The medical social workers stated that cancer survivors were more confident about their future after counseling or group programs. When cancer survivors listened to others’ vivid experiences of FCR and shared their concerns with the medical social workers, they began to gain a tangible sense of the future. These experiences reduced their vague distress or anxiety, and enabled them to manage their lifestyles and health.

“It naturally led to improving their daily routines or health. For example, it helped cancer survivors to think about how much they were willing to change their drinking behaviors, eating habits, work-out, and so on. Furthermore, they contemplated how they could make changes to improve their health. Afterwards, they were relieved from the vague fear that they had before.” (Participant 05)

#### 3.3.4. Informed in advance

Providing information in advance was effective in dealing with FCR among cancer survivors. Many cancer survivors did not recognize the existence of FCR before the intervention. Throughout the intervention, they gradually comprehended that FCR was a natural experience in their cancer journey.

“There were several cancer survivors saying ‘Oh, I wish someone had told me about this earlier.’” (Participant 06)

#### 3.3.5. Cancer survivors’ responses to medical social work interventions

The geographic location of cancer hospitals was related with cancer saviors’ responses to the interventions as well as their level of expressing FCR. Because of their geographical advantage, cancer survivors who resided in metropolitan areas had more opportunities to participate in various programs at tertiary or university cancer hospitals. Thus, these cancer survivors were more familiar with expressing FCR than those living in provincial areas. On the other way, it was not comfortable for cancer survivors in provincial areas to expose their psychological status. Similarly, the medical social workers had difficulties discussing coping skills with those living in provincial areas.

“I saw that cancer survivors in provincial areas had difficulties describing and coping with their fear. They would be more familiar with ‘Act first, think later.’. … It seemed that cancer survivors in metropolitan areas had already exercised expressing themselves. Unfortunately, cancer survivors living here (a provincial area) seemed to be unfamiliar with that practice.” (Participant 12)

### 3.4. Internal challenges to medical social work intervention

As the medical social workers intervened in cancer survivors’ FCR, they faced various challenges within the realm of their profession, including role conflicts in cancer hospitals and a lack of educational resources. Since addressing FCR was less prioritized because of their heavy workload, the medical social workers experienced challenges regarding providing medical information on FCR and delivering psychological interventions including CBT. However, they supported proactive efforts to deal with FCR among cancer survivors.

#### 3.4.1. Issues in professional roles

The medical social workers tended to face the limitations of their professional roles during their interventions for FCR in cancer survivors. Fear and cancer recurrence are associated with psychological and oncological issues, respectively. Thus, a few medical social workers had difficulty explaining FCR to cancer survivors from an oncological perspective.

“We are medical social workers. So, we cannot appropriately give them (cancer survivors) a medical-based explanation on the spot…” (Participant 09)

#### 3.4.2. Issues in implementing CBT

The medical social workers employed various counseling skills to deal with FCR in cancer survivors. Among various counseling methods, CBT is recognized as one of the most effective interventions for various psychological and behavioral issues, including depression and anxiety [22, 23]. A few medical social workers also agreed that CBT could be an effective intervention to alleviate FCR among cancer survivors. However, it remained challenging to implement CBT due to various limitations (i.e., time pressure, issues in applying special groups, lack of specific CBT guidelines for FCR, and differential competency among medical social workers).

“I think that some medical social workers would have difficulties implementing CBT. First, I think that this (CBT) should be commonly applied to FCR in cancer survivors by medical social workers. Subsequently, the CBT for FCR is to be manualized or standardized. As you know, this process is quite tough owing to the diversity of cancer survivors and situations… In my personal experiences, there are several limitations to implementing CBT…” (Participant 06)

#### 3.4.3. Lack of educational resources

The medical social workers pointed out a lack of educational resources regarding FCR. In reality, practical guidelines for FCR are unavailable in South Korea. Considering the imperativeness of FCR, practical guidelines for FCR should be developed. Moreover, the medical social workers suggested that the guidelines for FCR should include a wide range of content, such as assessment tools, practical skills, and various interventions. In the process of developing the guidelines, the knowledge and evidence of medical social work interventions for FCR need to be collected and organized. Finally, the guidelines for FCR should be disseminated to medical (cancer) social workers.

“If I have a valid measurement, I can easily and precisely assess my clients’ (cancer survivors’) FCR. As well, assessment can help me establish an appropriate plan for interventions… If the guidelines suggest proper interventions depending on clients (cancer survivors) or situations, we (medical social workers) are very willing to utilize it… In order to develop the guidelines for FCR, it is necessary to collect and organize the practice knowledge from medical social workers. We should also display tentative evidence of our interventions in the guidelines.” (Participant 09)

#### 3.4.4. Proactive intervention at cancer hospitals

All of the medical social workers emphasized that cancer hospitals and medical social workers should endeavor to provide information and support regarding FCR to cancer survivors. As such, a collaboration between medical and non-medical professionals should be facilitated at cancer hospitals.

“Any kind of intervention for FCR is necessary. Probably, an oncologist should refer cancer survivors in need to relevant programs on time… We should not wait for the cancer survivors’ request for help. Before cancer survivors seek help, we must approach them first. We have to introduce various resources to our clients (cancer survivors) in advance, even when they (cancer survivors) do not need them at the moment.” (Participant 02)

### 3.5. External challenges in medical social work intervention

During the interventions for FCR, the medical social workers discovered challenges that mainly occurred outside of their professional scope at cancer hospitals. Because cancer survivorship care can be provided in various ways and places, medical social work interventions for FCR should be expanded from cancer hospitals to families and communities. Thus, building resources and systems between cancer hospitals and the community is essential to address FCR in cancer survivors.

#### 3.5.1. FCR and cancer survivors’ families

The medical social workers found that cancer recurrence affected both cancer survivors and their families because it was associated with family dynamics. For this reason, they suggested that it was necessary to pay attention to FCR among cancer survivors’ families as well as that of cancer survivors.

“It is important to look at their families as well as cancer survivors. There are many cases in which family members feel more anxious or are perturbed (about cancer recurrence) even though the cancer survivors seem untroubled.” (Participant 05)

#### 3.5.2. Limited range of interventions at cancer hospitals

The medical social workers at cancer hospitals were hindered from addressing FCR in cancer survivors by the boundary of interventions. Most cancer survivors return home or to the community after active cancer treatment; medical social workers mainly serve cancer survivors at cancer hospitals. For this reason, the medical social workers encountered boundary issues in their interventions for FCR.

“I am sure that cancer survivors should freely expose their FCR any time… However, I sometimes wonder how much and long a cancer hospital can or should take care of their FCR…” (Participant 02)

#### 3.5.3. Developing resources and systems in the community

As mentioned above, most cancer survivors stay in the community after active cancer treatment, although they visit tertiary or university cancer hospitals for regular treatment and testing. According to the medical social workers’ experiences, it is unrealistic for cancer survivors to visit tertiary cancer hospitals only for resolving FCR. Therefore, it is necessary to develop community resources that provide various services (e.g., counseling and educational programs) to cancer survivors in need. In particular, the cancer-based facilities in South Korea (i.e., cancer education or survivor support centers) can play a role of dealing with survivors’ psycho-social issues including FCR in communities. Furthermore, the medical social workers proposed an integrated system that could enhance cancer survivors’ access to community resources.

“It would be better if cancer education centers or cancer survivor support centers regularly run these programs so they can monitor and provide services for those in need.” (Participant 01)

“If cancer hospitals are unavailable, experts should be dispatched to place where cancer survivors can easily visit. For example, community welfare centers or welfare offices could be a gatekeeper that provides them some help.” (Participant 11)

## 4. Discussion

From the interviews with the medical social workers, this study extracted five themes from their professional experience with cancer survivors’ FCR. Overall, these extracted themes can be discussed in terms of the internal and external aspects of medical social work intervention.

### 4.1. Internal implications for medical social work intervention

The internal implications of medical social work interventions are as follows: (1) intake process, (2) assessment and service plan, (3) intervention methods, and (4) educational resources. First, this study delineated how the medical social workers approached FCR in cancer survivors during the intake process. After establishing rapport with medical social workers, cancer survivors began to express their FCR, especially at the end of active cancer treatment. Therefore, it is important for medical social workers to build rapport with cancer survivors in a timely manner. Because cancer survivors rarely articulate “recurrence,” a medical social worker should motivate them to express complicated feelings, including FCR, in the intake process.

Second, this study reaffirms the importance of comprehensive assessment and individualized interventions for FCR among cancer survivors. FCR among cancer survivors was intertwined with a variety of characteristics, such as age, having a child(ren), caregiving responsibilities, and other cancer-related issues [24]. Therefore, FCR should be assessed from a holistic perspective that includes cancer issues (i.e., type, grade, and stage of cancer). Based on a comprehensive assessment, medical social workers should plan and provide individualized interventions for cancer survivors. It is well known that an individualized intervention contributes not only to facilitating cancer survivors’ compliance with treatment procedures but also to improving the medical outcomes of cancer treatments [25].

Moreover, this study discovered an interesting relationship between cancer survivors’ FCR and economic status. Although it differed from context to context, cancer survivors who were financially stable or in difficulty tended to exhibit FCR [26–28]. Indeed, Gormley et al. (2021) reported that moderate to severe FCR was relatively prevalent among cancer survivors in the middle or upper class [29]. The medical social workers in this study assumed that the differences in spare time and economic status in cancer survivors were associated with the level of expressing FCR. Cancer survivors who are financially stable can afford to participate in various psychological resources (e.g., counseling, education, programs, self-help groups, etc.). Hence, they have more opportunities to expose their feelings, including FCR. By contrast, cancer survivors with financial burdens had difficulties accessing these resources. Therefore, medical social workers should pay attention to not only the active FCR in cancer survivors but also to the latent FCR in those who are inaccessible to psycho-social resources for various reasons.

Third, this study proposes the ways in which medical social workers effectively intervene in FCR among cancer survivors. Although the medical social workers reported that basic counseling skills were effective and useful in addressing FCR in cancer survivors, they recognized the need to employ more advanced assessment tools and rigorous interventions [10]. For example, CBT is recommended as a highly effective intervention for FCR among cancer survivors [30, 31].

Fourth, this study suggests the development of educational resources regarding FCR in South Korea. In Canada and Australia, the guidelines for FCR were developed and disseminated to healthcare professionals such as oncologist and nurses [32–34]. Although an auxiliary guideline for FCR was recently developed in South Korea, it has not been extensively acknowledged among Korean medical social workers, and its contents should be further elaborated. Specifically, the medical social workers in this study suggested that guidelines for FCR should cover a wide range of practical contents (i.e., measures and thresholds of FCR, counseling skills and interventions, and basic knowledge of oncology). Hence, efforts to develop and disseminate guidelines for FCR contribute to reducing the disparity of psycho-social services in healthcare professionals and cancer hospitals as well as to promoting medical social workers’ competency in addressing FCR among cancer survivors [35].

### 4.2. External implications for medical social work intervention

Although the implications discussed above are appropriate within the realm of the medical social work profession, they are insufficient for a broader healthcare system. As this study explored the medical social workers’ experience with FCR in cancer survivors, it uncovered critical themes in the external aspect of medical social work. From a broader perspective, this study discusses the external implications in the following ways: (1) collaboration, (2) network, and (3) system integration.

First, this study upholds the collaborations of healthcare professionals to effectively address FCR among cancer survivors. In this study, the medical social workers as non-medical healthcare professionals were limited to advising FCR in cancer survivors on the solid basis of medical and treatment information; by contrast, medical professionals such as oncologists and nurses tended to concentrate on cancer treatments more than cancer survivors’ psychological issues. Likewise, Berrett-Abebe et al. (2018) also asserted that non-medical professionals should also be knowledgeable about oncology information (i.e., diagnosis, prognosis, type of treatment, and its long-term effects) to effectively address FCR that cancer survivors’ experience after cancer treatment [7]. In addition, the medical social workers in this study mentioned that an inter-specialty referral between medical and non-medical healthcare professionals should be more facilitated in interventions for FCR among cancer survivors.

Second, this study suggests building multidimensional networks considering the level of care and geographic locations of cancer hospitals. Akin to other countries, most tertiary or university cancer hospitals in South Korea are located in metropolitan areas. Thus, cancer survivors residing in provincial areas have more drawbacks when visiting tertiary or university cancer hospitals than those in metropolitan areas [36]. Moreover, they rarely visit tertiary cancer hospitals to seek help only for psychological issues. Considering these geographical barriers, building networks among cancer hospitals in metropolitan and provincial areas can be an alternative to effectively address cancer survivors’ psychological issues, including FCR. For instance, the Australian government piloted “The Healthy Living after Cancer” (HLaC), which was a partnership program to alleviate these geographic barriers [37]. This partnership program, which provided 6-month telephone-based lifestyle services led by healthcare professionals to cancer survivors in rural areas, demonstrated its effectiveness in improving participants’ physical and psychological symptoms along with FCR [37]. As such, building networks among cancer hospitals can empower cancer survivors to cope with various physical and psycho-social issues during their cancer journey [34, 38].

Finally, this study recommends the establishment of a community-based intake system (or gatekeeper) to coordinate various services for cancer survivors. As mentioned above, this study found that a particular group of cancer survivors was challenged with access to psychological services owing to individual and organizational issues. Therefore, a community -based intake system can pave an avenue for cancer survivors to engage in cancer treatments and cancer-related resources, and create a platform that links cancer hospitals and community resources. For these reasons, several countries have adopted an integrated system for cancer survivors (e.g., Italy, Spain, the U.S., the U.K., Australia, etc.) [39–41]. Therefore, the National Cancer Survivorship Center in South Korea, which manages general survivorship-related care at local level, can also expand its roles in building a community-based intake system for cancer survivors in need.

### 4.3. Limitations

There are several limitations to this study. Overall, snowball sampling may provoke credibility and dependability issues. Thus, the results of this study might show the experiences of medical social workers who were more interested in cancer survivors than others. Moreover, the opinions and appreciations of the medical social workers on other healthcare professionals and community resources in this study should be revisited in future studies. Furthermore, this study inherently contains issues of transferability owing to the study period and sampling. FCR has not widely and actively been considered among Korean medical social workers. Hence, our results should be carefully interpreted in the context of such medical social workers.

## 5. Conclusion

To the best of our knowledge, this study is the first trial to explore medical social workers’ experiences with FCR among cancer survivors in South Korea. From the interviews with 12 medical social workers, a wide range of phenomena and issues in medical social interventions for FCR among cancer survivors were identified. Based on these findings, we suggest internal and external implications with regards to medical social work to address FCR among cancer survivors.

In-depth interviews with 12 medical social workers enabled the present study to delineate a wide range of phenomena and issues in medical social work interventions for FCR among cancer survivors. First, this study observed when and how FCR among cancer survivors emerged during the preliminary stage of medical social work interventions. Second, the results of the study illustrated how medical social workers dealt with FCR in cancer survivors during their interventions. Third, the responses of cancer survivors to medical social work interventions for FCR were evaluated. Finally, this study discovered the internal and external issues underlying the engagement in FCR among cancer survivors.

## Supporting information

S1 File Consolidated criteria for reporting qualitative research (COREQ) checklist. (DOC)

## Abbreviations

FCR: Fear of Cancer Recurrence
TNBC: Triple-Negative Breast Cancer
CBT: Cognitive Behavioral Therapy
HLaC: Healthy Living after Cancer
